# TAGLN2-Regulated Trophoblast Migration, Invasion and Fusion are Impaired in Preeclampsia

**DOI:** 10.3389/fcell.2022.810633

**Published:** 2022-02-23

**Authors:** Hao Wang, Xiaowei Zhang, Chunfeng Liu, Shengfu Chen, Xinyang Liu, Shangrong Fan

**Affiliations:** ^1^ Department of Obstetrics and Gynecology, Peking University Shenzhen Hospital, Shenzhen, China; ^2^ Department of Obstetrics and Gynecology, Sun Yat‐Sen Memorial Hospital, Guangzhou, China; ^3^ Shenzhen Key Laboratory on Technology for Early Diagnosis of Major Gynecological Diseases, Shenzhen, China

**Keywords:** preeclampsia, transgelin-2, cell invasion, cell fusion, E-cadherin

## Abstract

Preeclampsia (PE) is a serious disease during pregnancy that affects approximately eight million mothers and infants worldwide each year and is closely related to abnormal trophoblast function. However, research on placental trophoblast functional abnormalities is insufficient, and the etiology of PE is unclear. Here, we report that the expression of transgelin-2 (TAGLN2) was downregulated in the placenta of patients with PE. In addition, a lack of TAGLN2 significantly reduced the ability of trophoblasts to migrate, invade and fuse. A co-immunoprecipitation (Co-IP) and microscale thermophoresis analysis showed that TAGLN2 bound directly to E-cadherin. A decrease in TAGLN2 expression led to a reduction in cleavage of the E-cadherin extracellular domain, thereby regulating the function of trophoblasts. In addition, we found that a reduction in soluble E-cadherin may also have an effect on blood vessel formation in the placenta, which is necessary for normal placental development. What’s more, the *in vivo* mouse model provided additional evidence of TAGLN2 involvement in the development of PE. By injecting pregnant mice with Ad-TAGLN2, we successfully generated a human PE-like syndrome that resulted in high blood pressure and some adverse pregnancy outcomes. Overall, the association between TAGLN2 and PE gives a new insight into PE diagnosis and treatment.

## Introduction

Preeclampsia (PE) is a serious disease during pregnancy that affects approximately eight million mothers and infants worldwide each year (Fisher, 2015; Burton et al., 2019). This complication is characterized by new onset hypertension (Croke, 2019) and may be accompanied by other serious symptoms, such as thrombocytopenia, renal insufficiency, impaired liver function, pulmonary edema and even new onset brain or visual impairment (American College of Obstetricians and Gynecologists, 2013; Poon and Sahota, 2019). Presently, the only clear treatment is delivery of the placenta, which therefore means delivery of the baby. Despite decades of research, a full understanding of the pathogenesis of PE remains incomplete, complicating the development of predictive biomarkers and targeted therapeutic strategies.

During human pregnancy, chorionic cytotrophoblast (CTB) progenitor cells follow one of two differentiation pathways, becoming either syncytiotrophoblasts (STBs) or extrapyramidal CTBs (Red-Horse et al., 2004). Both CTBs and STBs play key roles in the development and maintenance of a successful pregnancy. Some of the CTBs differentiate into extravillous trophoblasts (EVTs) with a high infiltration capacity (Phipps et al., 2019), and these cells migrate and invade the maternal decidua and the adjacent third of the myometrium during the interstitial invasion. EVTs also penetrate the uterine spiral artery wall, replacing the endothelium, destroying vascular smooth muscle, and transforming these blood vessels into a large diameter, low resistance catheter (Sheridan et al., 2019). The invasion of shallow CTBs and insufficient repair of the spiral artery are markers of early-onset PE (Brosens et al., 2011).

STBs also play an important role in the placenta. The fusion of monocyte trophoblasts to form nonproliferating multinucleated trophoblasts is essential for normal placental function. STBs participate in various processes, including hormone production, nutrient transport, and immune tolerance, and are closely related to the occurrence of PE (Yockey and Iwasaki, 2018). There are many factors that regulate trophoblast fusion, including cytokines, hormones, protein kinases, and transcription factors (Redman and Staff, 2015), but the mechanisms are still poorly understood. The fusion of CTBs can be induced by agents such as forskolin8 and inhibited by hypoxic conditions (Alsat et al., 1996). The fusion of trophoblasts is characterized by a decrease in the level of the cell adhesion molecule E-cadherin (Coutifaris et al., 1991), which is closely related to the fusion, migration and invasion functions of different kinds of cells (Aghababaei et al., 2015; Padmanaban et al., 2019).

Transgelin-2 (TAGLN2) is a 24 kDa protein that contains a calponin homolog (CH) domain and crosslinks with actin. TAGLN2 binds to actin to facilitate the formation of cytoskeletal structures, and is always linked with E-cadherin to drive the various biological process (Huang et al., 2021; Rasool et al., 2021). TAGLN2 is involved in cell transformation and regulating cell morphology (Shapland et al., 1993; Dvorakova et al., 2014) and mediates regulation of the epithelial-mesenchymal transition (EMT) by downregulating E-cadherin expression (Kim et al., 2018). Recently, many studies have revealed the important role of TAGLN2 in tumor progression and found that TAGLN2 is dysregulated in a variety of malignant tumor types, including lung cancer (Jin et al., 2016), bladder cancer (Yoshino et al., 2011), breast cancer (Dvořáková et al., 2016) and uterine cervical carcinoma (Yakabe et al., 2016), and is associated with tumor proliferation, migration and even angiogenesis. Our previous research found that TAGLN2 is highly expressed in embryonic trophoblasts and is associated with the adhesion of embryos. The invasion and migration abilities of human trophoblast cells were suppressed after TAGLN2 knockdown. The direct binding of TAGLN2 and actin has also been proved (Liang et al., 2019). In addition, recent studies have found that in PE patients, TAGLN2 secreted by CTB vesicles is significantly reduced (Tan et al., 2014). However, the effect of TAGLN2 on placental trophoblasts and the effect of TAGLN2 downregulation on placental-derived diseases such as PE remains unclear.

In this study, we first confirmed that the expression of TAGLN2 in preeclamptic placentas at the protein level was decreased compared with that in age-matched control placentas, and we explored the effect of TAGLN2 on trophoblast migration and invasion. Because TAGLN2 plays a role in cell adhesion, we examined TAGLN2 expression in BeWo cells during syncytialization and tested the hypothesis that TAGLN2 facilitates the fusion of trophoblasts. Then, we confirmed that the effect of TAGLN2 on invasion and syncytialization occurs by directly binding and regulating E-cadherin. Finally, we confirmed *in vivo* that a phenotype resembling PE is induced after knocking down TAGLN2 in pregnant mice.

## Methods and Materials

### Collection of Placental Tissues

Placental tissue was collected according to previously described standards and methods (Peng et al., 2019). Placental tissue was obtained from PE patients (*n* = 10) and uncomplicated pregnant patients (*n* = 10) who were admitted to Peking University Shenzhen Hospital. PE was diagnosed with a new systolic blood pressure ≥140 mmHg and/or diastolic blood pressure ≥90 mmHg after 20 weeks of gestation in the absence of preexisting nephropathy-associated proteinuria and/or essential hypertension. Patients with other major pregnancy complications were excluded. Early pregnancy villus tissue (*n* = 5) was collected from women whose pregnancies were legally terminated at 6–10 weeks of gestation. These women were medically healthy and had no history of spontaneous abortion or ectopic pregnancy. All placental tissues were collected according to a protocol that was approved by the Research Ethics Committee of Peking University Shenzhen Hospital. Written informed consent was obtained from all patients. The clinical characteristics of the patients are shown in Table 1.

**TABLE 1:** Clinical characteristics of participants.

Category	Control (n = 10)	Preeclampsia (n = 10)	*p* Value
Patient age (year)	30.05 ± 5.45	32.17 ± 4.88	0.074
Gestational age (weeks)	38.01 ± 1.59	36.64 ± 2.42	0.061
BMI at delivery (kg/m^2^)	28.51 ± 5.78	30.81 ± 4.09	0.089
Proteinuria (g/24 h)	0 ± 0	3.18 ± 1.84	<0.001
Systolic blood pressure (mmHg)	110.7 ± 13.9	155.5 ± 15.0	<0.001
Diastolic blood pressure (mmHg)	70.88 ± 6.7	95.44 ± 9.85	<0.001
Neonatal birth weight (g)	3,340.55	2,660.37	0.038
Nulliparity	4 (40%)	6 (60%)	0.371

BMI, body mass index (in kg/m^2^); BP, blood pressure. Values are mean ± standard deviation.

### Immunohistochemistry

The villus and placental tissues were washed with phosphate-buffered saline (PBS) and fixed overnight with 4% paraformaldehyde (PFA) at room temperature. Next, the samples were dehydrated, embedded in paraffin, and cut into 4 mm thick sections. The sections were deparaffinized, rehydrated, and then microwaved in 10 mM sodium citrate (pH 6.0) for 20 min to recover antigens and blocked with 3% H_2_O_2_ for 10 min. The sections were incubated overnight at 4°C with mouse primary antibodies against TAGLN2 (1:200; NBP1-89722; Novus, Colorado, United States). The following day, after the sections were washed with PBS, a secondary antibody (ab121146, Abcam, Cambridge, United Kingdom) conjugated with horseradish peroxidase (HRP) was applied and incubated for 20 min at room temperature, and then a diaminobenzidine solution was used to visualize the staining.

### Western Blot Analysis

Protein extracts were prepared from placental tissues and cells using radioimmunoprecipitation assay (RIPA) buffer (Beyotime P0013, Jiangsu, China) supplemented with protease and phosphatase inhibitors cocktail (PPC1010, Sigma-Aldrich, Darmstadt, Germany). For the western blot (WB) analysis, cell lysates (40 μg total protein) were electrophoresed by 10% SDS-PAGE and electrotransferred to a hydrophobic polyvinylidene fluoride membrane. After transfer, the membrane was blocked in TBST (0.15 M NaCl, 0.01 M Tris-HCl and 0.1% Tween-20, pH 7.4) containing 5% milk for 1 h and then washed thoroughly with TBST. The membrane was incubated with primary antibody (0.4 μg/ml; NBP1-89722; Novus, Colorado, United States). overnight at 4°C. The following day, the membrane was washed three times in TBST and incubated with the appropriate HRP-conjugated secondary antibody (0.4 µg/ml; ab121146; Abcam, Cambridge, United Kingdom) for 1 h at room temperature. Finally, the membrane was detected using an enhanced chemiluminescence detection system.

### Cell Culture and Small Interfering RNA (siRNA) Transfection

HTR8/SVneo cells (ATCC No. CRL-3271) were cultured in phenol red RPMI 1640 medium (Thermo Fisher Scientific, Waltham, MA, United States), and BeWo cells (ATCC No. CCL-9) were cultured in phenol red F12 medium (Thermo Fisher Scientific). Cells were grown at 37°C in 5% CO_2_. All cultures were supplemented with 10% fetal bovine serum (FBS, Thermo Fisher Scientific) and 100 U/ml penicillin-streptomycin (Thermo Fisher Scientific).

SiRNAs targeting the TAGLN2 gene and the negative control siRNA were purchased from Shanghai GenePharma. Transient transfection of cells with siRNA was performed with Lipofectamine 3,000 transfection reagent (Thermo Fisher Scientific) according to the manufacturer’s instructions. The TAGLN2 siRNA sequence was 5′-GGU​UAC​AGA​UGG​GCA​CCA​ATT-3′, and the negative control siRNA sequences was 5′-GCG​CGC​TTT​GTA​GGA​TTC​G-3′, as previously published as previously published (Liang et al., 2019).

### Cell Fusion Assays

BeWo cells were treated with forskolin (25 μM) for 72 h to induce cell differentiation. Cell fusion was quantified in BeWo cell cultures by microscopic analysis of E-cadherin (Proteintech, Shanghai, China) at cell-cell borders. Briefly, cell fusion was defined as at least three nuclei surrounded by cell membranes, as identified by continuous E-cadherin immunofluorescence staining (E-cadherin antibody: 1:50; 20874-1-AP; Proteintech, Shanghai, China); the nuclei were visualized with DAPI. Five random images were taken per well, and the proportion of fused cells was calculated as the number of nuclei in the multinucleated cell aggregates (>3 nuclei per aggregate) divided by the ratio of the total number of nuclei per field of view obtained by the ×40 objective.

### Immunofluorescence Staining and Confocal Microscopy

The cells were fixed in 4% PFA in PBS (pH 7.4) for 30 min at room temperature. Next, the cells were permeabilized in 0.5% Triton X-100 for 30 min and blocked in a 1% solution of bovine serum albumin (BSA, Sigma) for 30 min. The cells were then incubated with the primary antibody (1:100–1:200) overnight at 4°C. The following day, the cells were washed three times in PBS containing 0.1% Tween-20 and 0.01% Triton X-100 and transferred to a suitable mixture of fluorescent secondary antibodies (1 µg/ml; ab231303; Abcam, Cambridge, United Kingdom) for 1 h at room temperature. The nuclei were then stained with 10 μM DAPI for 10 min, and the cells were washed three times. Finally, the cells were mounted on glass slides. Observations were made under a confocal laser scanning microscope (Carl Zeiss 710, Jena, Germany).

### Three-Dimensional (3D) Z-Stack Imaging

E-cadherin was imaged as a z-series at 1 μm intervals, and the entire E-cadherin structure was captured by 3D confocal z-stacking. The CZI file was imported, and the E-cadherin status was analyzed using Zen program software.

### Colony Formation Assay

Cells were seeded in 10 mm plates at a density of 500 cells/plate. After 10 days of incubation at 37°C, the cells were fixed in 4% PFA for 30 min and stained with Giemsa staining solution for 20 min. Colonies consisting of more than 50 cells were counted and photographed.

### Real-Time Cell Proliferation, Migration and Invasion Assays

Cell proliferation, migration and invasion abilities were examined using a Real-Time Cell Analysis (RTCA) system (RTCA DP Instrument; ACEA Biosciences, Inc., United States). For continuous monitoring of cell proliferation, cells were seeded into RTCA E-plates at a density of 5 × 10^3^ cells per well, and the electrical impedance in each well was continuously measured for 50 h. The change in electrical impedance is expressed as a cell index, which is a parameter of cell proliferation.

Cells were treated with 10 µg/ml mitomycin for 3 h in CIM-16 plates to inhibit cell proliferation before migration and invasion experiments were performed. For invasion experiments, a layer of Matrigel (BD Biosciences) was also added to the upper chamber of the plate for 1 h. Next, 3 × 10^4^ cells were inoculated and monitored for 80 h. The sensor impedance is defined as the cell index.

### Tube Formation Assay

Matrigel with reduced growth factor was placed in a 96-well cell culture plate (50 μL/well) and incubated for 30 min. HTR8/SVneo cells (3 × 10^4^/100 μL) were seeded in Matrigel-coated wells. Tube formation was allowed to occur for 6–8 h before the medium was aspirated from the well, and 100 μL of a 2 μM calcein AM (Abeam) solution was added to each well of the 96-well plate and incubated for 15–30 min. Tube formation was observed under an inverted microscope. Calcein AM-labeled cells were photographed using a fluorescence inverted microscope at an excitation wavelength of 488 nm.

### Microscale Thermophoresis Analysis

A microscale thermophoresis (MST) analysis was performed according to a previously described protocol (Xie et al., 2018). Briefly, purified recombinant proteins were labeled with the RED-NHS protein labeling kit (NanoTemper Technologies, Munich, Germany) according to the manufacturer’s protocol. E-cadherin protein (Proteintech) was then incubated with a constant concentration (1 mM) and 2-fold serial dilutions of TAGLN2 protein, which purified as previously published (Liang et al., 2019), in MST-optimized buffer (50 mM Tris-HCl, pH 7.4, 150 mM NaCl, 10 mM MgCl_2_ and 0.05% Tween-20). An equal volume of the binding reaction was mixed by pipetting and incubated for 15 min at room temperature. The mixture was sealed in a standard treated glass capillary and loaded into an instrument (Monolith NT.115; NanoTemper). The measurement times were as follows: fluorescence for 5 s before, MST for 30 s, fluorescence for 5 s after and a delay of 25 s. The measurements were collected at 60% MST power.

### Immunoprecipitation and E-Cadherin Cleavage Assay

For the E-cadherin cleavage test, equal volumes of preconcentrated (*via* 10 kDa concentrators; Millipore, Darmstadt, Germany) conditioned medium from cells seeded at 1.5 × 10^6^ cells per plate were tested by WB analysis with anti-E-cadherin antibody (Cell Signaling Technologies, Danvers, MA, United States; 32A8 extracellular domain).

For immunoprecipitation experiments, cells were washed in ice-cold PBS and incubated in RIPA buffer (Beyotime P0013, Jiangsu, China). A total of 500 μg of whole cell lysate was immunoprecipitated using 2 μg of anti-TAGLN2 antibody. Protein A/G agarose bead conjugation followed by washing in ice-cold RIPA buffer was used to purify and wash target proteins.

### Establishment of TAGLN2-Deficient Mice During Pregnancy

Eight-to 12-week-old CD-1 female mice weighing 25–35 g from the Charles River (Beijing, China) were mated with age-matched male mice. Pregnant mice were randomly assigned to three groups [gestation day 14.5, adenovirus (Ad)-control (Ctrl), *n* = 5; Ad-TAGLN2, *n* = 5; and gestational day 18.5, Ad-Ctrl, n = 5]. Adenovirus carrying shRNA targeting mouse TAGLN2 was purchased from Genechem (Shanghai, China). Age-matched nonpregnant (NP) mice were divided into two groups (Ad-Ctrl, *n* = 5; Ad-TAGLN2, *n* = 5). The presence of a vaginal plug was considered gestational day 0.5. On gestational day 8.5, the pregnant mice were injected with Ad-Ctrl or Ad-TAGLN2 (2 × 10^9^ PFU, 100 μL) through the tail vein. systolic blood pressure was monitored by a tail cuff photoplethysmography BP-2000 Series II instrument (Visitech Systems, Apex, NC, United States) daily from gestational day 6.5–18.5. The mice were euthanized on gestational day 18.5 to collect placentas to examine the expression of TAGLN2 and weights of fetal and placental.

### Statistical Analysis

Proliferation and invasion curves were analyzed by a 2-way analysis of variance (ANOVA) using SPSS 23.0 software (IBM, Armonk, NY, United States). Student’s t test was used for all other data comparisons *via* GraphPad Prism six software (La Jolla, CA, United States). The means and standard deviations were plotted. All experiments were performed three times unless otherwise indicated.

## Results

### Trophoblastic TAGLN2 is Downregulated in PE Placentas

**FIGURE 1:**
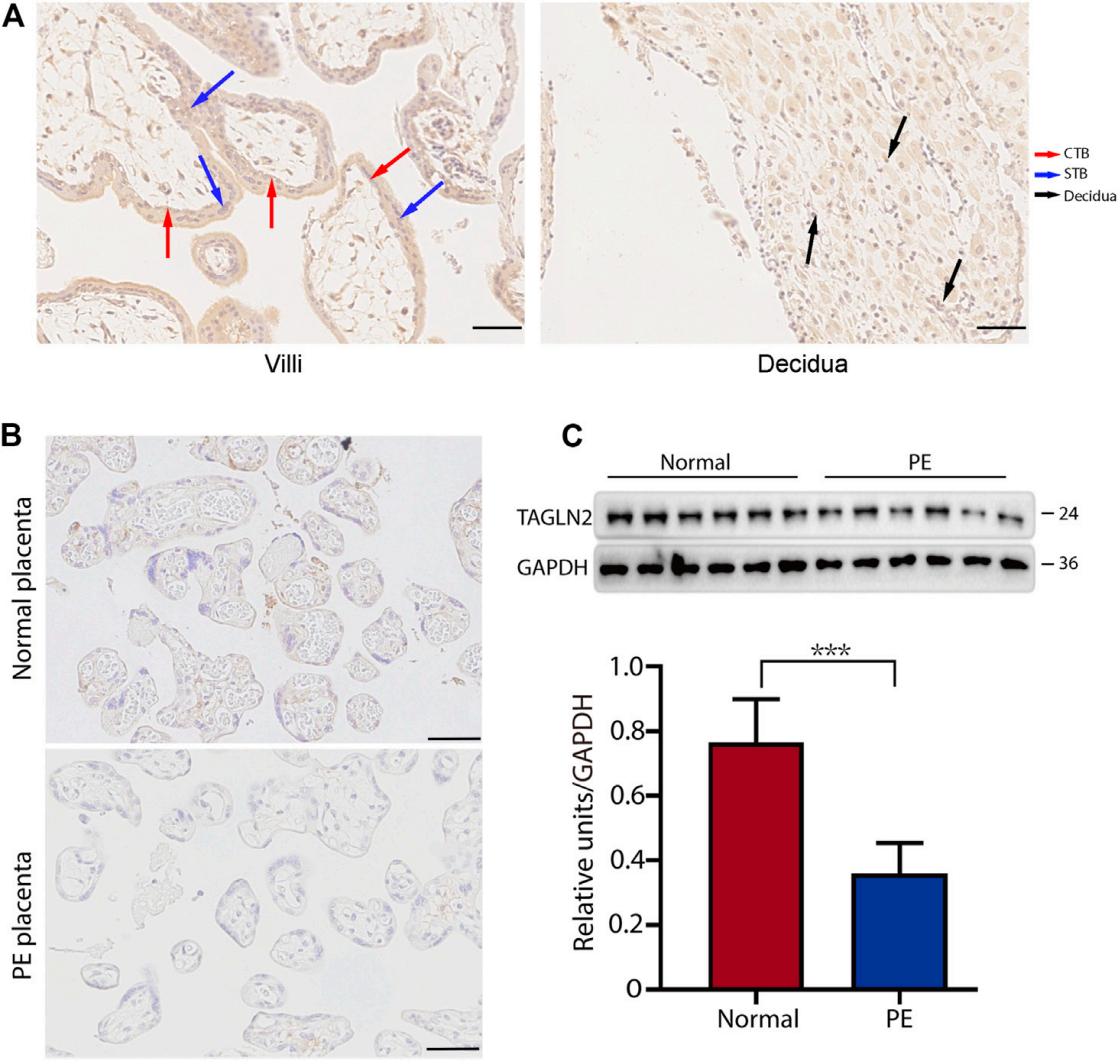
Expression of TAGLN2 in placentas. **(A)** IHC staining of TAGLN2 in the villi and decidua of first-trimester human placentas. The arrows are pointing to the staining areas that showing the expression of TAGLN2. Scale bar, 100 μm. **(B)** IHC staining of TAGLN2 in normal and PE placental tissues. Scale bar, 100 μm. **(C)** The expression of TAGLN2 in placental tissues was determined by WB analysis in PE and normal control placental tissues. The data are presented as the mean ± standard error of the mean (SEM). ***p < 0.001.

We first used immunohistochemistry (IHC) to assess the expression level of TAGLN2 in the human placenta in early pregnancy. The results showed that TAGLN2 was expressed in different trophoblast subtypes, including villous STBs and CTBs and interstitial EVTs (iEVTs) in the decidua (Figure 1A). To further compare the difference in TAGLN2 expression between PE and normal gestational age-matched placentas, we performed IHC to detect the level of TAGLN2 in both groups. The results showed that the staining intensity in both groups was weaker than that in early pregnancy, indicating that TAGLN2 expression may gradually weaken as pregnancy progresses, but the expression in the PE group decreased more significantly than that in the normal group and almost no staining was detected (Figure 1B). The WB results further revealed that the expression level of TAGLN2 was significantly lower in the PE placenta than in the normal placenta (Figure 1C). Collectively, these results suggest that the downregulation of TAGLN2 in trophoblasts is associated with PE placentas.

### Loss of TAGLN2 Inhibits Trophoblast Migration and Invasion

**FIGURE 2:**
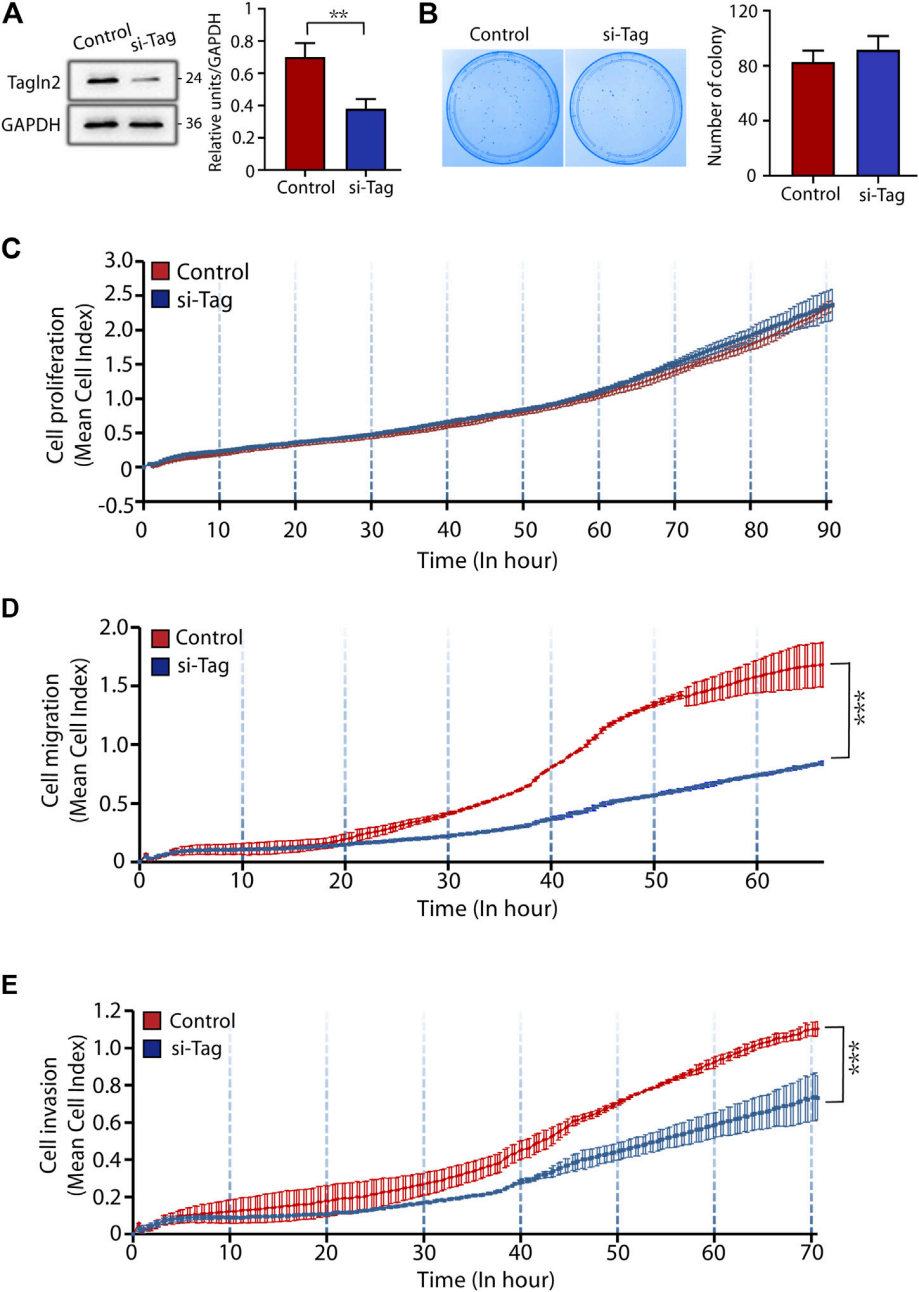
TAGLN2 regulates trophoblast migration and invasion. **(A)** Validation of TAGLN2 knockdown by WB analysis. Equal amounts of normal control and si-TAGLN2 cell lysates were subjected to WB analysis with a TAGLN2 antibody. Experiments were performed at least three times. The values are represented as the mean ± SEM. ***p < 0.001. **(B)**. Cell colony experiment of the two groups of cells. Experiments were performed at least three times. The values are represented as the mean ± SEM. **(C)** Proliferation cell index (CI) of the 2 cell lines based on the RTCA real-time monitoring system. The data were analyzed using 2-way ANOVA. **(D)** Migration CI of the 2 cell lines based on the RTCA real-time monitoring system. The data were analyzed using 2-way ANOVA. **(E)** Invasion CI of the 2 cell lines based on the RTCA real-time monitoring system. The data were analyzed using 2-way ANOVA.

To explore the role of TAGLN2 in trophoblast function, we used the BeWo cell line. Cells treated with nontargeted scrambled siRNA were used as controls, and the efficiency of TAGLN2-siRNA knockdown was confirmed by WB analysis (Figure 2A). We first explored the function of TAGLN2 in cell proliferation. Colony formation experiments showed that TAGLN2 knockdown did not have a significant effect on the number or size of colonies formed (Figure 2B); therefore, we used an RTCA and found that the dynamic monitoring of the two groups was basically the same (Figure 2C).

Next, we transfected cells with small interfering (si)-TAGLN2 and si-control and seeded the cells into CIM plates. After 70 h of culture, the migratory ability of cells was significantly decreased in the si-TAGLN2 group compared with the si-control group (Figure 2D). For the invasion assay, a layer of Matrigel was added to the upper chamber of the CIM plates to simulate the extracellular matrix and provide a barrier to cell invasion. As shown in Figure 2E, the invasive ability of cells in the si-TAGLN2 group was much lower than that in the control group. These data indicate that TAGLN2 is important for the migration and invasion of trophoblasts.

### Loss of TAGLN2 Inhibits Trophoblast Fusion

**FIGURE 3:**
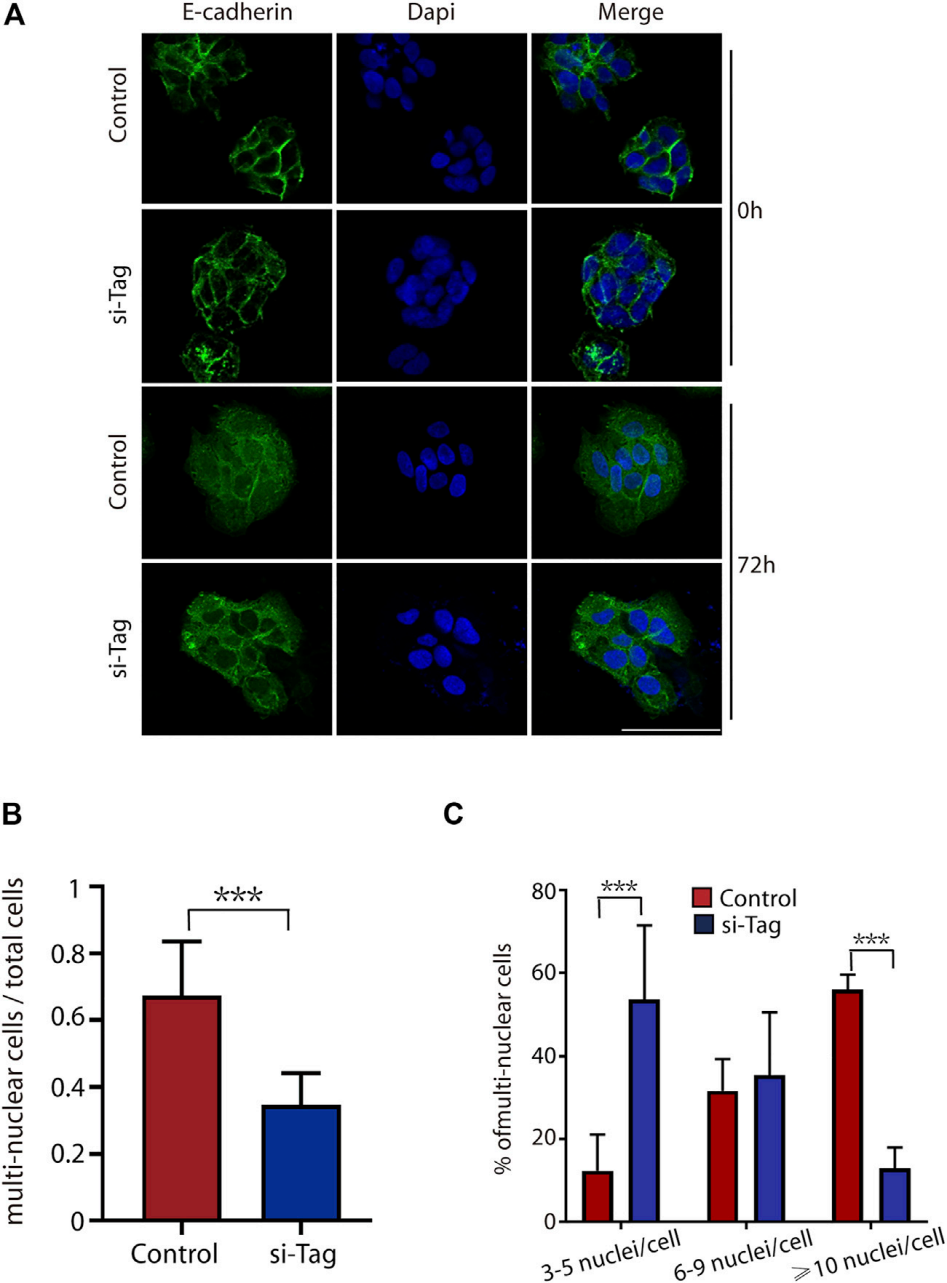
TAGLN2 regulates trophoblast fusion. **(A)** Representative immunofluorescence images of si-control and si-TAGLN2 BeWo cells probed with antibodies against E-cadherin after 0 and 72 h of culture in the presence of forskolin8. DAPI-stained nuclei are labeled blue. Bar = 100 μm. **(B)** The proportion of multinuclear cells (≥3 nuclei per cell) to the total number of cells after 72 h of culture in the presence of forskolin8. **(C)** The proportion of multinucleated cells containing three to five nuclei, six to nine nuclei, or ≥10 nuclei to the number of multinucleated cells treated with forskolin8 for 72 h. The data are presented as the mean ± SEM. ***p < 0.01.

For the STB fusion analysis, we transfected BeWo cells with si-control and si-TAGLN2 and then used forskolin8 to induce cell fusion. Transfection of cells with control siRNA did not affect cell differentiation, and the cells fused into multinuclear structures and displayed E-cadherin reorganization by 72 h (Figure 3A). In contrast, silencing TAGLN2 resulted in profound intercellular retention of E-cadherin (Figure 3A). The loss of TAGLN2 led to a reduction in the proportion of multinucleated structures (Figure 3B). Consistent with this finding, the number of multinucleated structures containing ≥10 nuclei was obviously decreased in TAGLN2-silenced cells, but they were observed in control siRNA-transfected trophoblasts (Figure 3C).

### TAGLN2 Directly Binds to E-Cadherin and Directs Ectodomain Shedding of E-Cadherin

**FIGURE 4:**
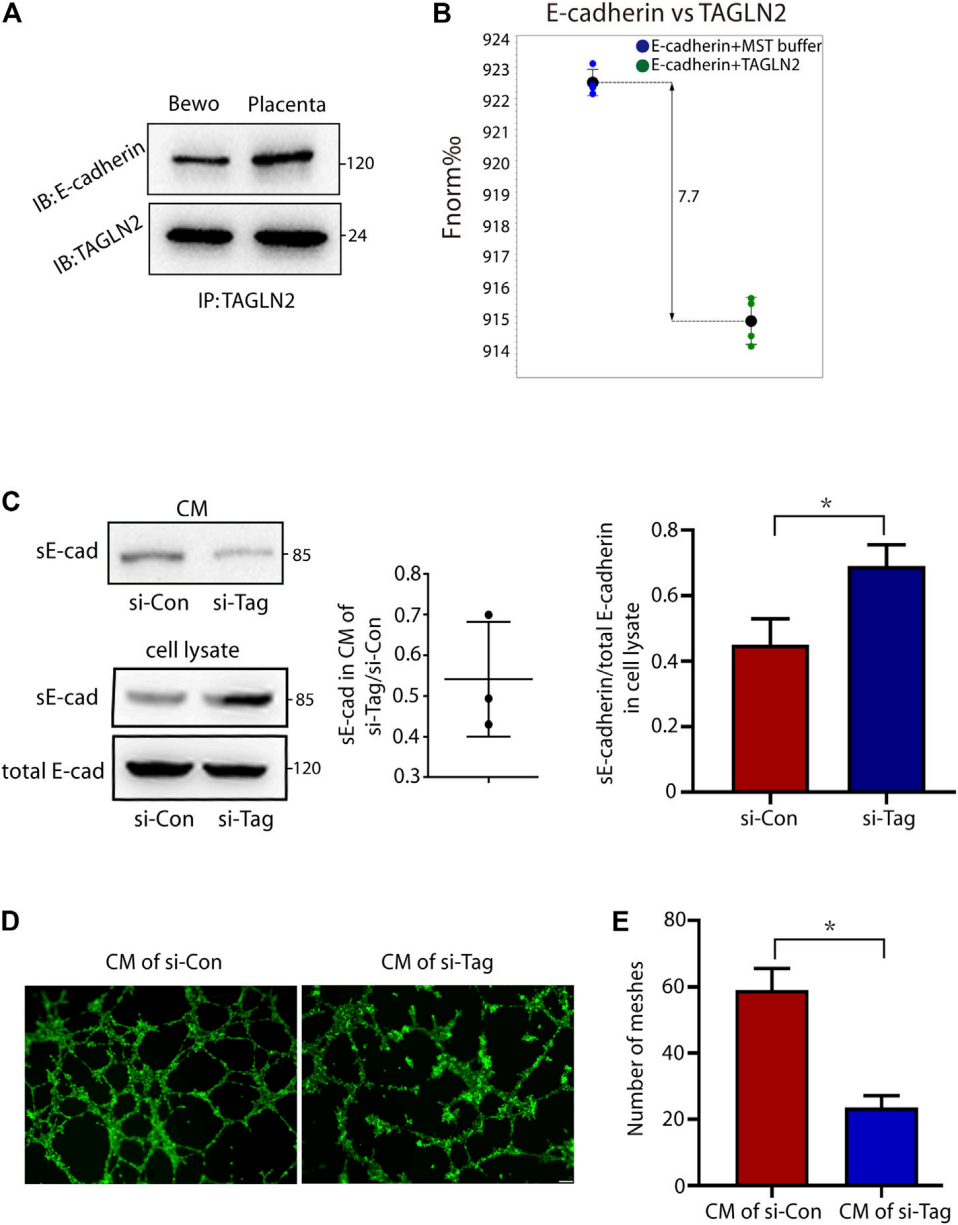
TAGLN2-directed ectodomain shedding of E-cadherin and disturbed endothelial cell function. **(A)** Total cellular proteins from placental tissue and BeWo cells were immunoprecipitated with anti-TAGLN2 antibody, separated by SDS-PAGE and immunoblotted with E-cadherin antibody. **(B)** MST results of TAGLN2 and E-cadherin analysis. Normalized fluorescence (Fnorm%), which equals to F1/F0 (F1: fluorescence after thermodiffusion; F0: initial fluorescence or fluorescence after T-jump). **(C)** Immunoblots showing E-cadherin in conditional medium (CM) or whole cell lysate from control and si-TAGLN2 BeWo cells. The gray levels of the gel image were measured and the ratio of statistical graphs. **(D)** Matrigel endothelial-like tube formation was examined with HUVECs cultured in CM from control and si-TAGLN2 BeWo cells. Scale bar, 100 μm. **(E)** The number of meshes was calculated and is expressed in pixels. All experiments were performed at least three times. All data are represented as the mean ± SEM. *p < 0.05.

As an adhesion junction molecule, E-cadherin is an important protein in cell migration, invasion and fusion. Therefore, we next explored whether TAGLN2 could interact with E-cadherin and affect the normal function of E-cadherin and immunoprecipitated total protein extracts of placental tissues and BeWo cells with an anti-TAGLN2 antibody. WB analyses of the immunoprecipitants indicated that the specific antibody did coimmunoprecipitate the E-cadherin protein (Figure 4A), suggesting that both TAGLN2 and E-cadherin either physically interact or are colocalized in trophoblasts. Next, we used the MST assay to further confirm that TAGLN2 and E-cadherin can directly bind with each other (Figure 4B).

The extracellular domain of E-cadherin is an important functional area, and disruption of E-cadherin in TAGLN2-downregulated cells suggests the involvement of proteolytic shedding. To assess this possibility, we examined conditioned medium and whole cell lysates from si-NC- and si-TAGLN2-transfected cells. The WB results showed a decrease in the level of sE-cadherin in the conditioned medium of si-TAGLN2-transfected cells (Figure 4C), while in the cell lysate, the level of sE-cadherin in the si-TAGLN2 transfection group was increased compared to the control group, but the total E-cadherin level was almost the same in the two groups (Figure 4C). These observations indicate that TAGLN2 directly binds to E-cadherin and participates in regulating proteolytic shedding of the E-cadherin extracellular domain.

A recent study found that sE-cadherin is a potent inducer of angiogenesis (Tang et al., 2018). To further understand the pathogenic role of TAGLN2 in PE, we performed a tube formation assay with HUVECs in the presence of BeWo cell in conditional medium, and the results showed that the HUVECs treated in conditional medium from the si-TAGLN2 group exhibited smaller regular tube areas and formed fewer tubes compared to cells treated with conditional medium from the si-NC group (Figures 4D,E).

### TAGLN2-Deficient Mice Demonstrated Compromised Placentation and a PE-like Phenotype

**FIGURE 5:**
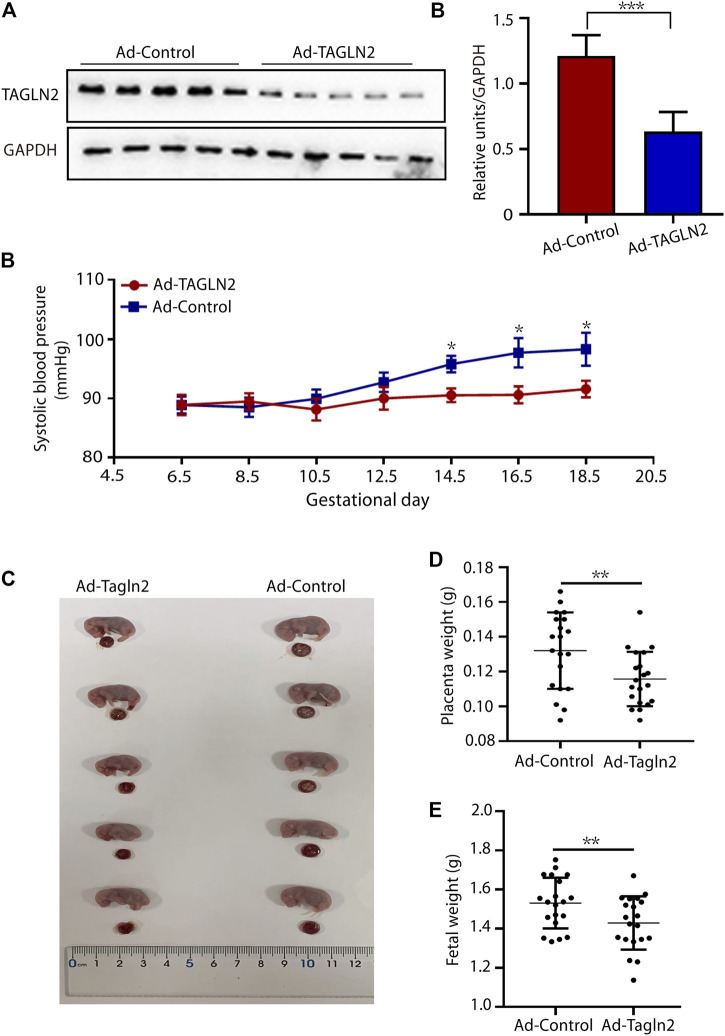
TAGLN2-deficient mice have PE-like phenotypes. **(A)** WB analysis of TAGLN2 in placentas collected from Ad-Ctrl- (*n* = 5) and Ad-TAGLN2-injected (*n* = 5) mice on gestational day 18.5. **(B)** systolic blood pressure was monitored daily in the Ad-Ctrl (*n* = 4) and Ad-TAGLN2 (*n* = 4) groups on gestational day 6.5 and 18.5. **(C)** Fetal mouse and placentas in the two groups on gestational day 18.5. **(D)** Placental weight on gestational day 18.5. (Ad-Ctrl, *n* = 20 pups from five dams; Ad-TAGLN2, *n* = 20 pups from five dams). **(E)** Fetal weight on gestational day 18.5 (Ad-Ctrl, *n* = 20 pups from five dams; Ad-ACTN4, *n* = 20 pups from five dams). The data are expressed as the means ± SEM. *p < 0.05, **p < 0.01, ***p < 0.001.

To further validate our *in vitro* findings, TAGLN2-deficient mice were generated by an intravenous injection of TAGLN2-interfering adenovirus on gestational day 8.5. TAGLN2 expression was significantly downregulated in the Ad-TAGLN2 group on gestational day 18.5 (Figure 5A). To evaluate the final pregnancy outcomes, we examined systolic blood pressure and placental and fetal weight in adenovirus-infected pregnant mice that were euthanized on gestational day 18.5. As shown in Figure 5B, the systolic blood pressure of the Ad-TAGLN2 group was higher than that of the Ad-Ctrl group. Placental and fetal weights on gestational day 18.5 were both lower in TAGLN2-deficient mice than in Ad-Ctrl mice (Figures 5C–E). These results indicate that TAGLN2 deficiency impairs placentation and induces a PE-like phenotype *in vivo*.

## Discussion

PE has been well characterized molecularly. However, the significance of many of the changes in gene expression remains unclear. TAGLN2 was investigated in this study, which showed a lower expression level in PE placentas than normal placentas and was mainly associated with CTBs and STBs. The association between TAGLN2 and PE is properly mediated by smooth muscle. During normal pregnancy, cytotrophoblasts emigrate from the chorionic villi and invade the maternal decidua, and further deeply invade the spiral arteries within the uterine wall. Then the cytotrophoblasts migrate upward along these vessels, retrograde to replace the resident portions of the smooth muscle wall. While in the PE cases, the infiltration of cytotrophoblasts in the spiral artery is incomplete, which will result in a much smaller number of cytotrophoblasts in blood vessels and retains part of endothelial cells, and lead to defective angiogenesis (Redman and Sargent, 2005; Fisher, 2015). Hence, the expression of receptors and ligands expressed by vascular smooth muscle cells or endothelial cells, like the smooth muscle cell marker transgelin, will not up-regulated as in normal pregnancy. On the other hand, the development of smooth muscle cells during gestation is stage-specific. The myofibroblast, which is the similar cell type of smooth muscle cells developed in the follicular phase, its immunohistochemical expression of thrombomodulin is increased in severe PE (Bosco et al., 2005). While for the cytoplasmic filaments type that smooth muscle cells developed during the luteal phase in early pregnancy, its dense bodies are sparse in PE (Katabuchi et al., 2003). Therefore, as the differentiation of the smooth muscle plays a key role during the occurrence of PE, the transgelin as a biomarker of differentiated smooth muscle should also link with PE.

In the current study, we also found that the migration and invasion capacities of trophoblasts were markedly decreased when TAGLN2 was knocked down. Since the shallow invasion of trophoblasts in the placenta leads to a decrease in the volume of maternal blood flowing into the intervillous space and causes several negative consequences including PE (Saito and Nakashima, 2014), TAGLN2 should be an important gene that associated with the aetiology of PE. Interestingly, TAGLN2 has been reported to be upregulated in many tumors and regarded as a proto-oncogene (Bischof et al., 2001; Ferretti et al., 2007). For example, a high expression of TAGLN2 in tumor-derived lung cancer endothelial cells has been associated with clinical stage, tumor size, and tumor development in lung cancer tissues (Jin et al., 2016). In addition, the inhibition of TAGLN2 in human cervical squamous cell carcinoma (SCC) and uterine SCC cells has been shown to significantly inhibit tumor growth and invasion (Fukushima et al., 2011). These consistent findings might result from the similar migration and invasion abilities of trophoblasts and tumor cells, but further research is needed due to they are different types of cells. Moreover, our previous study has been confirmed the important role TAGLN2 played in human and mouse human trophoblast cells. We also explained that TAGLN2 regulated the invasion and adhesion of trophoblast by promoting actin polymerization (Liang et al., 2019). Hence, TAGLN2 might influence the aetiology of PE in a similar way. What’s more, our *in vivo* mouse model provided additional evidence of TAGLN2 involvement in the development of PE. By injecting pregnant mice with Ad-TAGLN2, we successfully generated a human PE-like syndrome that resulted in high blood pressure and some adverse pregnancy outcomes. After understanding these, TAGLN two could be a biomarker for the early diagnose of PE.

Syncytialization of trophoblasts and the formation of STBs is another key molecular event in pregnancy, and it plays a vital role in fetal-maternal nutrient exchange and steroid hormone synthesis. This syncytialization is essential for successful placental development and fetal growth (Kliman et al., 1986; Gerbaud and Pidoux, 2015). The syncytium of the preeclamptic placenta is thin, vacuolated and discontinuous (MacLennan et al., 1972) and exhibits a defect in cell fusion function (Langbein et al., 2008). Here, we observed that knockdown of TAGLN2 significantly reduced the syncytialization of BeWo cells, and it was directly interacting with E-cadherin in both placental extracts and BeWo cells. Silencing TAGLN2 appeared to inhibit E-cadherin extracellular domain shedding and reduce the strength of cell adhesion in tissues, resulting in enhanced cell motility, which plays an important role in trophoblast fusion. Conversely, cleavage of the extracellular domain of E-cadherin promotes the Wnt/β-catenin signaling pathway. Therefore, the downstream transcription factor GCM1, which is a key factor for trophoblast fusion, may be regulated (Matsuura et al., 2011). In addition, E-cadherin activates multiple pathways [e.g., epidermal growth factor receptor (EGFR) and insulin-like growth factor receptor (IGFR)] and molecules, such as matrix metallopeptidases (MMPs), and promotes cell migration (Beavon, 2000) and invasion (Symowicz et al., 2007; Brouxhon et al., 2014). Moreover, the direct binding of TAGLN2 and actin has been confirmed by our previous study (Liang et al., 2019), which may result in the further linkage of E-cadherin to diver the various biological process. Thus, enhancing the interaction between TAGLN2 and E-cadherin or directly promoting the expression of TAGLN2/E-cadherin may be the new idea of PE treatment.

A recent study found soluble E-cadherin (sE-cadherin) to be a potent inducer of angiogenesis (Tang et al., 2018), which is associated with the principal pathogenesis of PE (Walsh, 2006). In our experiments, we also verified that a reduction in sE-cadherin levels in the culture medium reduced the efficiency of HUVEC tube formation. In addition, another study found that the expression level of E-cadherin in implanted placental tissue was significantly reduced, while the level of sE-cadherin was increased (Duzyj et al., 2015), indicating that sE-cadherin is closely related to placental abnormalities. Therefore, decreased levels of sE-cadherin in the placenta may also be an important factor in PE pathogenesis.

However, our study has some limitations. First, we did not explore the specific mechanism by which TAGLN2 causes abnormal expression levels of sE-cadherin. We hypothesize that there are several possible mechanisms. First, the binding of E-cadherin to intracellular proteins is very important for E-cadherin structure and function (Chen et al., 2016; Advedissian et al., 2017); thus, the binding of TAGLN2 and E-cadherin may change the structure and properties of E-cadherin, thereby affecting cleavage of the E-cadherin extracellular domain. The second possibility is the impact of the combination of the two proteins on the downstream pathway. Some factors in the downstream pathway, such as EGF (Grabowska et al., 2012), may also affect E-cadherin cleavage. Third, current studies have shown that sE-cadherin is also secreted by exosomes (Tang et al., 2018), and placenta-derived exosomes are synthesized by STBs via the lysosomal pathway (Pillay et al., 2017); therefore, STB fusion disorder may in turn further enhance the reduction in sE-cadherin secretion. In addition, recent studies have shown that TAGLN2 activation causes relaxation of respiratory smooth muscle (Yin et al., 2018), but the direct effect of TAGLN2 on vascular smooth muscle and blood pressure is still unclear. The increase in blood pressure observed in our experiments may also be directly caused by maternal vasoconstriction. However, considering the characteristics of the PE pregnant mouse model and the lack of effective methods to specifically interfere with the expression of placental molecules, the specific reason for the effect of TAGLN2 knockdown on blood pressure needs further research.

In conclusion, our present study reports that TAGLN2 levels are decreased in the trophoblasts of pregnant women with PE. Our *in vitro* results confirmed that the downregulation of TAGLN2 expression inhibits trophoblast migration, invasion, and syncytialization and mediates endothelial cell dysfunction by regulating E-cadherin. Furthermore, downregulation of TAGLN2 expression *in vivo* induces PE symptoms and impairs spiral arterial modulation in a mouse model. Our findings indicate that TAGLN2 may be a potential biomarker for the early diagnosis of PE and that increasing the actions of TAGLN2 might be a specific and safe therapeutic strategy for treating patients with PE.

## Data Availability

The raw data supporting the conclusion of this article will be made available by the authors, without undue reservation.
